# The Role of Galectins as Modulators of Metabolism and Inflammation

**DOI:** 10.1155/2018/9186940

**Published:** 2018-05-21

**Authors:** Monica Fengsrud Brinchmann, Deepti Manjari Patel, Martin Haugmo Iversen

**Affiliations:** Faculty of Biosciences and Aquaculture, Nord University, 8049 Bodø, Norway

## Abstract

Galectins are *β*-galcotosid-binding lectins. The function of galectins varies with their tissue-specific and subcellular location, and their binding to carbohydrates makes them key players in several intra- and extracellular processes where they bind to glycosylated proteins and lipids. In humans, there are 12 identified galectins, some with tissue-specific distribution. Galectins are found inside cells and in the nucleus, cytosol, and organelles, as well as extracellularly. Galectin-1, -2, -3, -4, -7, -8, -9, and -12 can all induce T-cell apoptosis and modulate inflammation. In the context of metabolic control and loss of the same in, for example, diabetes, galectin-1, -2, -3, -9, and -12 are especially interesting. This review presents information on galectins relevant to the control of inflammation and metabolism and the potential to target galectins for therapeutic purposes.

## 1. Introduction

Saccharides are key energy molecules in metabolic pathways. They are also used to modify proteins and lipids to make glycolipids and glycoproteins, that are important in intracellular and extracellular processes. It is therefore not surprising that lectins that bind sugar groups are important modulators of many processes and key functional players in others. Galectins are proteins that bind *β*-galactosides such as N-acetyllactosamine present in N-linked and O-linked glycoproteins. They are involved in the control of, among others, pre-mRNA splicing, and in the control of apoptosis, cell cycle, cell division, metastasis, and diabetes [1–4]. They can bind to and agglutinate bacteria, and some galectins can kill bacteria directly without the activation of other factors, such as complement factors [5, 6]. In humans, genes for galectin-1, -2, -3, -4, -7, -8, -9, -10, -12, -13, -14, and -16 are found in GenBank (https://www.ncbi.nlm.nih.gov/genbank/ version 19.12.2017, 23.30.00). Even if carbohydrate binding is the classical galectin mode of action, galectins can also interact with other proteins in a carbohydrate-independent manner [7]. One or two galectin carbohydrate recognition domain(s) (CRD(s)) is/are present in the galectins. In prototype galectins (galectin-1, -2, -7, -10, -13, -14, and -16), the protein consists of the globular CRD, the galectin fold with two beta sheets. The chimera galectin-3 has a C-terminal CRD and an N-terminal tail, whilst the tandem repeat galectins (galectin-4, -8, -9, and -12) have two CRDs. Known galectin ligands are, among others, CD45, CD7, CD43, CD2, CD3, CD4, CD107, CEA, laminin and fibronectin, glycosaminoglycans, integrins, GM1 ganglioside, polypeptide HBGp82, glycoprotein 90 K/MAC-2BP, CA125 cancer antigen, and pre-B cell receptor (reviewed in [8]). Galectins can often be both negative and positive modulators of the same processes, suggesting plasticity in isoforms, posttranslational modification(s) or comolecules present, and/or differences in localization.

Processes such as inflammation and metabolism are tightly regulated and fine-tuned. Lack of control can lead to diseases such as diabetes; in fact, chronic, low-grade inflammation is seen in obesity, and this inflammation can lead to obesity-related insulin resistance and eventually type 2 diabetes mellitus [9]. In diabetes mellitus, a long-standing hyperglycemic state can give production of advanced glycation end products (AGEs) that bind to organic molecules and cause complications [10]. In obesity, inflammation is increased, and the number of activated (proinflammatory) macrophages, associated with insulin resistance in human obesity, increases in human fat tissues [11]. This accumulation and activation of macrophages in turn change the metabolism in white adipocytes, and obesity-related insulin resistance can be looked upon as a disease promoted by chronic inflammation in adipose tissues [12].

Galectin family members are molecules increasingly focused upon as regulators of cellular processes including metabolism and inflammation. This review will focus on the roles of galectins in metabolism and inflammation to elucidate if the galectins could be key regulators involved in the linking of both processes. The reviews' metabolism control of galectin part will be centred on diabetes and obesity, two processes where inflammation and metabolism regulation are important both in the development of imbalances and in upholding them.

## 2. Main Text

### 2.1. Galectin-1

Galectin-1 is a prototype galectin with one carbohydrate recognition domain. It is, among others, released from adipose tissues [13], stromal cells in the thymus, lymph nodes, endothelial cells [14], and placenta cells [15]. Apoptosis of activated (antigen-primed) T-cells is induced by galectin-1 in a CD45-dependent manner [14], and T-cell homeostasis can be regulated by galectin-1 through inhibition of clonal expansion and induction of apoptosis. Activated T-cell apoptosis induced by galectin-1 is caspase-8- and -9-dependent [16]. Interestingly, activated T-cells themselves can produce galectin-1 through MEK1/ERK, p38 MAP kinase, and p70S6 kinase signalling pathways, suggesting that this is an autocrine suicide mechanism used to terminate an effector immune response [17]. On the other hand, resting T-cells bind galectin-1, but apoptosis is not induced [14]. Galectin-1 is anti-inflammatory, and its mRNA is upregulated in the placenta in preeclampsia; this is suggested to be a fetal response to maternal systemic infection [15]. The anti-inflammatory activity of galectin-1 can be used to reduce allergic conjunctivitis, an inflammation of the conjunctiva and other parts of the eye. In an allergic conjunctivitis model in mice, externally administered recombinantly produced galectin-1 was an inhibitor of allergic reaction. Galectin-1 reduced IgE levels; however, this was only a slowing of the IgE response as, at 24 hours, galectin-1-treated mice had higher IgE levels than nontreated (allergy control) mice had. In spite of the higher IgE levels, clinical signs were reduced in galectin-1-treated mice [18].

Therapeutic administration of galectin-1 suppresses T-cell-dependent chronic inflammation in arthritis [19], in hepatitis [20], and in colitis [21], suggesting a broad potential for therapeutic use in inflammatory diseases.

Interestingly, in type one diabetes, T-cell-mediated autoimmunity destroys insulin-producing pancreatic *β*-cells. This process can be inhibited by galectin-1 *in vivo* [17]. Galectin-1 is suggested to have a role in improving glucose metabolism in obese people [22], and galectin-1 levels are higher in obese children than in normal-weight children [22]. Galectin-1 has a potential to be used as an inhibitor of inflammation-related diseases such as diabetes, and also inhibiting galectin-1 led to weight loss in diet-induced obese mice, where weekly injections with a galectin-1 inhibitor attenuated adipogenesis and lipogenesis and increased expression of proteins associated with thermogenesis and energy expenditure [23]. Hence, both stimulation and inhibition of galectin-1 can be of therapeutic value. Galectin-1 levels increase in eyes of patients with diabetes after accumulation of AGEs, and vitreous aspirates from eyes of patients with diabetic macular edema and later proliferative diabetic retinopathy have increased galectin-1 levels. AGE production induces IL-1*β* via ERK1/2, and PI3K signalling is involved in galectin-1 induction in these patients [24]. This increase in galectin-1 level could be a protective response to the increased AGE levels, similar to that seen in kidneys where galectin-3 is involved in clearance of AGEs [25].

### 2.2. Galectin-2

Galectin-2 is a prototype, single-CRD galectin, primary expressed in the gastrointestinal tract which can induce T-cell apoptosis through the caspase-3- and -9-dependent intrinsic apoptotic pathway where, among others, cytochrome c leaves the mitochondria. The apoptosis activation is independent of binding to the glycoprotein CD3 or CD7 [16]. In the presence of galectin-2, activated T-cell profiles are modulated to be dominated by Th2 [16].

Interestingly, in a search for genes associated with insulin resistance, a genotype of galectin-2, LGALS2 rs7291467, was stronger associated with changed fasting plasma glucose and serum insulin than other alleles were [26], and the same genotype which has a single-nucleotide polymorphism (3279C → T) in intron 1 of LGALS2 encoding galectin-2 was previously found to be significantly associated with myocardial infarction [27]. Galectin-2 binds to the proinflammatory cytokine lymphotoxin-*α* to regulate inflammation [27]. Galectin-2 is present in the pancreas, but not in islets—only in exocrine cells [28]. It would be interesting to see if galectin-2 present in the pancreas could influence inflammation and the production of insulin in beta cells in the nearby islet; however, we failed to find functional galectin-2 studies on diabetes or obesity.

### 2.3. Galectin-3

Galectin-3 has several alternative names. Galectin-3 was early characterised in the outer membrane of macrophages and named Mac-2 antigen [29]; found to be IgE binding and named *ε*BP [30]; named CBP35, carbohydrate-binding protein 35 from mouse 3T3 fibroblasts [31]; and named CBP30 as baby hamster kidney carbohydrate-binding protein [32].

Galectin-3 is the only chimera galectin; it has a C-terminal carbohydrate recognition domain as well as an N-terminal tail. Galectin-3 can, like galectin-1, induce T-cell apoptosis [33]; the N-terminal end and the CRD coordinate to induce signalling pathways leading to caspase-9 activation [34]. Inhibiting the *N*-terminal end with an antibody shuts down apoptosis stimulation [34]. When it comes to apoptosis, galectin-3 can be both antiapoptotic and proapoptotic. The antiapoptotic effect is dependent on phosphorylation of serine 6 in galectin-3 and the intracellular presence of galectin-3 [35]; phosphorylated, but not unphosphorylated, galectin-3 can be exported from the nucleus to the cytoplasm to act as an inhibitor of apoptosis by interacting with mitochondria to, among others, prevent cytochrome c release and caspase-3 activation [36, 37].

The N-terminal and C-terminal ends can both be involved in dimerization or possible oligomerisation of galectin-3, thought to be important for the function of the molecule [38, 39]. The binding properties of galectin-3 to their ligands are pH-dependent [40]; this raises the possibility that pH could be a contributing factor in determining galectin function in different locations including the cellular microenvironments. This is a property exploited in cellular sorting of galectin-3 in polarized Madin-Darby canine kidney cells [41].

Regulation of galectin-3 binding to its ligands is modulated by the phosphorylation state of serine in the N-terminal of the protein, where phosphorylation decreases binding and dephosphorylation increases binding [42]. Phosphorylation of tyrosine 107 can give the cleavage of galectin-3, and the ratio of phosphorylated/dephosphorylated galectin-3 can be used for the prognosis of prostate cancer as well as be a target for potential treatment [43].

Galectin-3 can be endocytosed by macrophages. Uptake of galectin-3 in classically activated M1 macrophages is carbohydrate-independent and mediated by N-terminal end binding, whilst uptake in alternatively activated M2 macrophages, as well as nonmacrophages, is carbohydrate-dependent and involves the C-terminal CRD [44]. In T-cells, galectin-3 is present at the cell surface associated with the TCR complex; it seems to inhibit uncontrolled T-cell activation and potentiates downregulation of TCR in T-cells [45]. Galectin-3 is observed in among others fibroblasts, chondrocytes, osteoblasts, osteoclasts, keratinocytes, Schwann cells and gastric mucosa. It is also found in endothelial cells in a number of tissues, and in immune cells such as neutrophils, eosinophils, basophils, mast cells, Langerhans cells and dendritic cells (cell types reviewed in [46]). It has a role in adipocyte proliferation, and obese mice have more galectin-3 in adipocytes than lean subjects have [47].

Interestingly, whilst galectin-1 can inhibit autoimmune diseases, the research focus on galectin-3's potential therapeutic use is in large focusing on inhibiting galectin-3. This is because galectin-3 promotes cancer and metastasis [1], and inhibition would hence have a great potential for therapeutic anticancer treatment.

Galectin-3 is however beneficial in other situations. It binds AGEs, glycated proteins, and lipids formed, among others, in diabetic patients, and galectin-3 knockout mice have accelerated glomerulopathy and higher renal/glomerular AGEs levels, suggesting that galectin-3 is involved in receptor pathways needed for AGE removal in kidneys [25]. AGEs are interesting as their receptor-mediated uptake led to cytokine production [48]. Galectin-3 can also be a receptor for lipoxidation end products (ALEs) [49]. Even if galectin-3 is beneficial to the kidney as AGE and ALE binders and thus inhibits inflammation, its proinflammatory effect can contribute to inflammation-produced kidney damage [50, 51]. The proinflammatory galectin-3 property has been linked to cardiac inflammation in obese patients [52] and to cardiac lipotoxicity (lipid deposits in the heart) and subsequent mitochondrial dysfunction affecting heart metabolism [53].

### 2.4. Galectin-4

Galectin-4 is a tandem-repeat galectin with two CRDs joined by a linker. Its main expression is in the gastrointestinal tract of healthy individuals where it has a role in control of intestinal inflammation [54]. In the intestine, galectin-4 interacts with activated T-cells through CD3 binding and promotes calpain-mediated T-cell apoptosis [54]. Importantly, it reduces proinflammatory cytokine production in the intestine mucosa in a colitis model [54]. However, it can also promote inflammation in the intestine by stimulating CD4^+^ T-cells to produce IL-6 [55].

Taken together, this suggests that galectin-4 could be important not only for ulcerative colitis but also for other inflammatory bowel diseases such as Crohn's disease. Galectin-4 has anticancer properties and has been shown to suppress colorectal cancer [56]; knockdown of it promotes tumorigenesis, and lower levels of galectin-4 expression were observed in inflamed precursor lesions of colorectal cancer [57]. Since T-cell response can help remove cancer cells, the fact that galectin-4 can promote mucosal T-cell apoptosis and also suppress colorectal cancer seems counterintuitive and suggests that there are mechanisms in colorectal inflammation and cancer prevention that warrant further studies. In addition, it is interesting that galectin-4 modulates inflammation, but there seem to be no studies focused on diabetes or other inflammation-dependent obesity diseases associated with galectin-4, suggesting that galectin-4 is not involved in modulation of metabolic diseases since it is mainly expressed in the intestine.

### 2.5. Galectin-7

Galectin-7 is a homodimeric prototype galectin with one CRD. It is mainly present in the epidermis [58] and can induce apoptosis of stimulated T-cells in a manner dependent upon caspase-1, -3, and -8, but not caspase-9 [16]. Galectin-7-deficient mice appear normal; however, when exposed to UVB irradiation, apoptosis is induced earlier and lasts longer than in wild-type mice [58]. This is surprising, since galectin-7 is proapoptotic [16]; however, the galectin-7-negative mice also show hyperproliferation of cells after UVB irradiation and after wounding [58], more in line with what one would expect when inhibiting the expression of a proapoptotic protein. Overexpression of galectin-7 in mice compromises the skin by leading to loss of cell junctions and defective skin repair [59]. Galectin-7's predominant expression in the skin suggests that it would not influence diabetes or obesity-related inflammation; however, one would suspect that it could play a role in skin diseases related to flawed control of the immune system. Hence, it is not surprising that, in the stratum corneum, galectin-7 is highly expressed in atopic dermatitis patients [60] and possible treatment of this and other skin diseases could target galectin-7.

### 2.6. Galectin-8

Galectin-8 is a tandem-repeat galectin with two CRDs joined by a linker [61]; it can exist in two splice variants with different linker lengths [62]. Altogether, six possible isoforms exist, three with two CRDs and three with one CRD [63]. Galectin-8 is expressed in the liver, kidney, cardiac muscle, lung, and brain [61] as well as in a number of different tumoral cells [63]. Galectin-8 induces apoptosis in T-cells through the expression of the death factor Fas ligand and gives caspase-mediated apoptosis [64]. Dimeric galectin-8 resulted in phosphatidylserine exposure in the outer bilayer of cells, something which normally happens during apoptosis activation; however, in the case of galectin-stimulated phospholipid redistribution, this occurred independently of apoptosis [65]. Galectin-8 is secreted under basal conditions from human microvascular endothelial cells and has an autocrine function in that extracellular galectin-8 stimulates the secretion of proinflammatory molecules, CXCL1 (GRO-*α*), GM-CSF, IL-6, and CCL5 (RANTES), from the endothelial cells [66]. Platelets can also express galectin-8 and be activated by the lectin in an N-terminal CRD-dependent manner [62]. Hence, both endothelial cells and platelets contribute to inflammation stimulated by galectin-8. Galectin-8 can also act proinflammatory by stimulating dendritic cells that secrete proinflammatory cytokines and stimulate antigen-specific T-cells [67]. Activation of neutrophils to produce superoxide is also stimulated by galectin-8 [68]. Galectin-8 is clearly involved in regulation of the immune system; however, we failed to find involvement of galectin-8 in diabetes or other inflammation processes induced by obesity.

### 2.7. Galectin-9

Galectin-9 is a tandem-repeat galectin with two CRDs joined by a linker sequence. It is expressed in the liver, small intestine, and thymus and in a lesser amount in the kidney, spleen, lung, and cardiac and skeletal muscle and in a low amount in reticulocytes and brain [69].

Galectin-9 is involved in T-cell selection in the thymus where it will induce apoptosis of CD4/CD8 double-negative or double-positive thymocytes and further in promoting naïve T-cell differentiation into Treg and by inhibiting naïve T-cell differentiation into T-helper 17 cells. Finally, it is involved through interaction with TIM3 (T-cell immunoglobulin domain and mucin domain protein 3) in apoptosis induction of CD4^+^ T-helper 1 (Th1) cells, Th17, cells and CD8^+^ cytotoxic T-cells. TIM3-negative Th2 cells do not go into apoptosis upon exposure to galectin-9, neither do TIM3-positive Treg cells (T-cells and galectin-9 reviewed in [70]). Taken together, this points to galectin-9 as an important molecule in the regulation of the immune system; in particular, the induction of apoptosis in Th1 and Th17 cells is interesting as activation of these cells is key in regulation of inflammation in, for example, autoimmune diseases. It also suggests that there is a need for research to investigate whether differential glycosylation of the cells can explain why not all TIM3-positive cells enter apoptosis when exposed to galectin-9, whether galectin-9 can act via other receptors [70], or whether there are differences in comolecules, costimulators, or cosuppressors yet to be described. The induction of apoptosis by galectin-9 is through the calcium-calpain-caspase-1 pathway; that is, cytochrome c is released from mitochondria and caspase-activated [71].

Galectin-9 expression in intestine epithelial cells is upregulated in the intestine in patients with food allergy. Mast cells stimulate galectin-9 expression by secreting tryptase, which in turn activate the proteinase-activated receptor 2 on the epithelial cells. Secreted galectin-9 will in turn activate dendritic cells [72]. In another mucosal surface, the airways, administered galectin-9 inhibited airway hyperresponsiveness and by binding to CD44 and inhibited hyaluronan attachment; galectin-9 inhibited Th2-associated inflammation in the airways [73].

As explained above, galectin-9 binds TIM3, a marker present on many immune cells. In early preeclampsia, peripheral lymphocytes, T-cells, cytotoxic T-cells, NK cells, and CD56^dim^ NK cells have reduced TIM3 levels, and an increased frequency of lymphocytic cells with positive galectin-9 expression is found. The lowered TIM3 levels could mean that the normal role of TIM3/galectin-9 in suppressing Th1 cells and secretion of IFN-*γ* and inducing apoptosis is compromised. One possible explanation is that galectin-9 cannot inhibit inflammation in a situation where TIM3 is downregulated [74]. In the maternal fetal interface, natural killer cells are involved in maternal tolerance of the fetus; the TIM-3/galectin-9 pathway is key to upholding the local tolerance by suppressing a unique NK cell subset's cytotoxicity toward trophoblasts [75]. These decidual NK cells are CD56-positive and CD16-negative cells that are important in immunomodulation in implantation and pregnancy [76]. The cytotoxic activity of decidual NK cells can be switched on; however, this is suppressed in normal pregnancies. Women with recurrent spontaneous abortion have an increased number of cytotoxic NK cells in the endometrium [77]. Interestingly, TIM3 inhibits degranulation of NK cells, and hence cytotoxicity toward trophoblasts, in a galectin-9-dependent way [75].

Galectin-9 is present in intestinal epithelial cells in low amount; however, the levels increase in patients with food allergy. Blocking galectin-9 in a mouse food allergy model inhibited the allergenic hypersensitivity status and Th2 polarization [72].

Galectin-9 is also expressed in adipose tissues, and in diet-induced obesity in mice, subcutaneous adipose tissue showed increased galectin-9 expression. Increased galectin-9 expression was also observed in both CD11c^−^ and CD11c^+^ macrophages in visceral adipose tissue compared to lean mice [13]. Galectin-9 is suggested to downregulate inflammation by modulating the interaction of macrophages with T lymphocytes via TIM3.

During obesity, fat can build up in the liver with nonalcoholic fatty liver disease as a result. The disease development is depending upon natural killer cells positive for TIM3; these cells will go into apoptosis when stimulated by galectin-9. Even if galectin-9 can also stimulate natural killer cell proliferation by increasing TIM3^+^ Kupffer cells' secretion of IL-15, exogenous administration of galectin-9 decreases the development of nonalcoholic fatty liver disease [78]. Similar results are also found in obesity-induced diabetes where a Th1 inflammation response is involved in development of diabetes. In mice, galectin-9, upregulated by injection of plasmid encoding galectin-9, inhibits the development of diabetes. This probably takes place through binding to TIM3, and galectin-9/TIM3 interacting reduced Th1 cell numbers in the spleen, pancreatic lymph node, and pancreas [79]. Galectin-9 could thus be a possible target for therapeutic strategies to reduce inflammation in obese atients to reduce diseases such as diabetes and fatty liver.

### 2.8. Galectin-10/Charcot-Leyden Crystal Protein

Galectin-10 is a prototype galectin present as dimers. When eosinophils are recruited to an inflamed site, they are observed to contain autocrystallizing Charcot-Leyden crystal protein, also named galectin-10. When eosinophils degranulate, the galectin-10 crystal deposited can stay in the tissues, in among others asthmatic patients, for extended periods of time. These crystals can induce inflammation in acute peritonitis and bronchitis [80]. Galectin-10 is present in large amounts in eosinophils and basophils, making up 7–10% of the total protein of eosinophils [81]. Galectin-10 is also expressed in CD4^+^CD25^+^ regulatory T-cells [82]; these T-cells are important in downregulation of antiself responses. Interestingly, inhibiting galectin-10 in activated CD25^+^ Treg cells restored Treg cell proliferative capacity and also overrode their suppressive function [82]. A subpopulation of eosinophils are regulatory eosinophils that can suppress T-cells, through a mechanism that are partly dependent upon galectin-10 [83]. Extracellular recombinant galectin-10 can also suppress T-cell proliferation [83]. Galectin-10 could be a human-specific galectin as it has not been found in other mammals [84]. For galectin-10, there is a need for more information on its molecular role in immunology. A recent study showed induction of IL-1*β* release upon Charcot-Leyden crystals/galectin-10 uptake by primary human macrophages. The IL-1*β* release was dependent upon activation of the NLRP3 inflammasome [80]. This indicates a key role for galectin-10 in inflammation, and further studies in this field hold a potential for novel new treatments of inflammation-dependent diseases.

### 2.9. Galectin-12

Galectin-12 is a tandem galectin with two CRDs where the C-terminal CRD has less homology with classic CRDs than the N-terminal CRD has; the latter has the classical lectin fold with two beta-sheets [85]. Galectin-12 is predominantly expressed in adipose tissue [86].

Differentiation of 3T3-L1 cells into adipocytes is inhibited by downregulation of galectin-12, and upregulation of galectin-12 induces G1 cell cycle arrest and apoptosis [87]. This suggests that galectin-12 has a central role in adipocyte turnover. Allyl isothiocyanate (AITC) reduced the expression of galectin-12 and could reduce the body weight of high-fat-diet-fed mice, further reducing the accumulation of lipid droplets in the liver, and white adipocyte size [88]. On the other hand, a restriction in calories fed increased galectin-12 mRNA levels and treatment of obese animals with troglitazone, a thiazolidinedione, increased galectin-12 expression and decreased adipose tissue size [86]. Hence, both reduced and increased expressions of galectin-12 decrease adipocyte size. The adipose tissues are key in the balance between triglyceride synthesis during energy surplus and lipolysis during energy needs. Interestingly, galectin-12 has a role in upholding this balance, and ablation of galectin-12 in mice shows that galectin-12 has a profound effect on lipid turnover and its removal induces lipolysis and decreases adiposity [89]. Galectin-12 is localised to lipid droplets where it inhibits lipolysis. The binding of galectin-12 to other molecules is probably not through the classical N-terminal CRD, as lactose does not influence the binding; however, the C-terminal nonclassical CRD is postulated to have less affinity for lactose [85] and could be involved in still-to-be-characterised ligand binding. An increase in lipolysis was not found in the liver and muscle in galectin-12-negative mice. This is not surprising as the main localisation of galectin-12 is in the adipose tissues [89]. Galectin-12 is inhibiting phosphorylation-dependent recruitment of hormone-sensitive lipase to lipid droplets probably by restricting the amount of the second messenger cAMP, and galectin-12-negative mice show increased phosphorylation of hormone-sensitive lipase by protein kinase A activated by cAMP. Galectin-12-deficient obese mice also show less insulin resistance/glucose intolerance than do wild-type obese mice so the insulin sensitivity and the glucose tolerance are increased [89]. Hence, galectin-12 is a possible target for therapies to reduce obesity and diabetes mellitus (type 2). Galectin-12 in the adipose tissues is also important for the inflammation state of the tissue as galectin-12 promotes inflammation [90].

Galectin-12 is expressed in macrophages, in addition to adipose tissues [90]. Interestingly, macrophages are infiltrating adipose tissues in obesity [11]. In adipose tissues of mice with diet-induced obesity, there is an increase in macrophages observed in adipose tissues before there is a substantial increase in insulin levels characteristic for systematic insulin resistance [12]. This increase in macrophages is not observed in the muscle, liver, lung, and spleen until very late (after 26 weeks on a high-fat diet) where macrophage gene CD68 was expressed in the liver [12]. It is suggested that the abnormal fat metabolism caused by the increasing adiposity is causing the macrophage accumulation [12]. The macrophages in the adipose tissues could release cytokines that lessen macrophage insulin sensitivity, which again would stimulate recruitment of more macrophages due to the metabolic changes [12]. The macrophages in adipose tissues are heterogenic. Classical M1 macrophages are proinflammatory, and the alternatively activated, M2 macrophages are anti-inflammatory. The proinflammatory macrophages are associated with insulin resistance [11]. In the liver, activation of Kupffer cells by the alternative activation pathway via the peroxisome proliferator-activated receptor delta counteracts obesity-induced insulin resistance [91]. Galectin-12^−/−^ macrophages showed lower phagocytosis of *E. coli* than did galectin-12^+/+^ macrophages, and galectin-12^−/−^ also promoted a M2 macrophage profile during macrophage activation [90] suggesting reduced inflammation when galectin-12 is not present. This is supported by the fact that galectin-12-negative mice fed a high-fat diet had less macrophage infiltrations into adipose tissue than control mice had, as well as lower cholesterol and triglyceride in serum [90]. Galectin-12 expression is linked to galectin-3 expression, and knockout of galectin-3 reduces adipose tissue expression of galectin-12 [92]. This suggests that galectin-3 inhibitors could not only target galectin-3 but also lead to reduced galectin-12 levels and hence decreased adipose inflammation. However, inhibition of galectin-12 will not change galectin-3 levels [90]; hence, targeting galectin-12 will not clinically help reduce galectin-3 effects.

### 2.10. Galectin-13, Galectin-14, and Galectin-16

Galectin-13, -14, and -16 were suggested to have placenta-specific expression predominantly in the syncytiotrophoblast, a primary site of metabolic exchange [93], and they are suggested to be important for pregnancy tolerance development. Galectin-13 is special in that the monomers in the homodimer are covalently linked by disulphide bonds [94] whilst other galectins interact noncovalently. Low galectin-13 levels in the third trimester are strongly correlated with preeclampsia [95], and low levels of galectin-13 expression in week 11 were observed in trophoblasts from residual samples of chorionic villus in women who later developed preeclampsia [96]. Galectin-13 can, as galectin-1, -3, and -9, induce apoptosis of activated T-cells, and it is suggested that this could be important to hinder maternal immune cells in attacking the fetus [93].

The extravillous trophoblast from the cervix of early pregnancy loss patients had a reduced level of galectin-14 [97, 98]; also, downregulation of galectin-14 has been found in preterm severe preeclampsia [15].

Galectin-13, -14, and -16 can induce apoptosis of T-cells important in the placenta [93].

The placental localisation of these galectins suggests they are not important for obesity-developed inflammation problems in the adult.

### 2.11. Clinical Potential of Galectins

Galectins have major roles in processes as diverse as cancer, obesity, diabetes, preeclampsia, and cardiovascular disease. Human galectin-1, -2, -3, -4, -7, -8, -9, and -12 are all interesting in an immunological perspective as they can induce T-cell apoptosis and modulate inflammation. This review has focused on the role of galectins in inflammation and metabolism, and in this perspective, the current knowledge points to galectin-1, -2, -3, -9, and -12 as especially interesting. For galectin-1 and galectin-9, an increase in the levels of the proteins could protect against diabetes, whilst for galectin-12 inhibition of the protein could be protective. In the case of galectin-3, the data are not as straightforward. In the case of galectin-2, we did not find diabetes or obesity-related studies, but galectin-2 modulates inflammation and its presence in the pancreas is interesting and its role in inflammation in the pancreas could be further studied.

In principle, there is hence a potential to treat diseases and disorders by inhibiting some galectins; for others, direct administration of galectin on surfaces or by injections could be beneficial, and for both groups, targeted decrease or increase, respectively, of the expression of the proteins could be therapeutic. The fact that galectins are involved in numerous cellular and intracellular processes, and the fact that there is conserved sequence homology between the galectins, could complicate treatment strategies. However, increased knowledge on galectins' site-specific functions, posttranslational modifications, and binding partners could make it possible to construct modified recombinant galectins as well as specific inhibitors [99]. To increase inhibitor specificity, work on designing new inhibitors and studying the inhibitors' interactions with different galectins [100] is important and promising.

Proof of concept of galectin inhibition and the use of administered galectin as treatments come from animal experiments described for the individual galectins above and in this section, and in a limited amount from clinical trials.

Intraperitoneal injection of galectin-1 prevents onset of hyperglycemia and reverse pancreatic beta cell autoimmunity in the pancreas in mice [101]. In addition, healing of pathological wounds in diabetic mice was accelerated by subcutaneous injection of galectin-1 [102]. Galectin-1 is implicated in tumor development [3] and in obesity [22, 23], and the galectin-1 inhibitor thiodigalactoside reduced the body weight gain in mice [23].

We did not find clinical studies in human targeting galectin-1 by the use of inhibitors or injections of the protein. However, a study using bevacizumab (antibody against vascular endothelial growth factor) and ipilimumab (antibody against cytotoxic T-lymphocyte-associated antigen 4) on patients with metastatic melanoma found that patients with therapeutic responses made antibodies against galectin-1, whilst a group of patients with reduced survival had increased circulating galectin-1 protein levels [103]. There is a wide interest in galectin-1 as a possible therapeutic target, and inhibitors of galectin-1, modified galectin-1, antibodies against galectin-1, and strategies for targeted delivery are currently investigated, and several companies, universities, and institutes hold patents for the use of these potential treatment strategies (thoroughly reviewed in [99]). Since both administration of galectin-1 and inhibition of it can be beneficial, studies need to be holistic and body weight, diabetes, and cancer should be monitored in further work.

Galectin-3 is a complex therapeutic target as it is involved in both inhibition and stimulation of inflammation. Treatment targeting galectin-3 must also take into consideration the fact that galectin-3 promotes cancer and metastasis [1] and that there is not a causal necessity between a protein being upregulated in a disease and the possibility to treat the disease by inhibiting the protein. This is clearly shown in a transgenic fibrotic cardiomyopathy model in mice, where galectin-3 was upregulated both at mRNA and protein levels; however, neither galectin-3 inhibitors nor galectin-3 knockout were effective in reversing cardiac fibrosis or inflammation [104]. This suggests that in fibrotic cardiomyopathy, at least in this mouse model, galectin-3 increase is a symptom, not an inducer of the disease. In a heart failure model in mice, however, proposed inhibition of galectin-3 with modified citrus pectin reduced myocardial inflammation, and reversed isoproterenol induced fibrogenesis that, untreated, led to left ventricular dysfunction [105]. It should be noted that even though pectins inhibit galectin-3-induced hemagglutination and cell interaction [106], a thorough *in vitro* study of plant-derived polysaccharides, including pectin, showed low inhibition or no inhibition at all of galectin-3 [107]. Therefore, the reduced myocardial inflammation effects of citrus pectin could be through other mechanisms than direct galectin-3 inhibition.

There are several inhibitors targeting galectin-3 (reviewed with a focus on patents in [108]). In humans, there are a few studies targeting galectin-3. The galectin-3 inhibitor GR-MD-02 (galactoarabino-rhamnogalacturonate; it also binds galectin-1 but with lower affinity) was used in a double-blinded study on subjects with nonalcoholic steatohepatitis with advanced fibrosis. The highest dose tested reduced fibrosis [109]. The same drug was used to study potential inhibition of inflammation in psoriasis subjects (only five subjects and no control group). Interestingly, patients infused with GR-MD-02 biweekly 13 times showed an average of >50% reduction in the Psoriasis Area Severity Index [110]. However, the lack of control subjects, the few subjects included, and the short time frame studied suggest that further studies must be conducted before one can conclude that the galectin-3 inhibitor has potential for treating psoriasis.

Galectin-9 promotes apoptosis of several cell types, including T-cells, in a TIM3-dependent manner [70] and hence modulates inflammation. In development of type 1 diabetes, insulin-producing beta cells in the pancreas are destroyed; this autoimmune destruction is dependent on Th1 cells. Interestingly, upregulation of galectin-9 by injection of a galectin-9 plasmid in mice significantly protected them from diabetes [79]. In another mouse study, injection of galectin-9 inhibited development of diabetes, and an antibody against TIM3 was at least as effective as galectin-9 injections in protecting against development of diabetes [111]. This strongly suggests that galectin-9 and molecules interacting with it such as TIM3 have key roles in diabetes and have a therapeutic potential. A study using prebiotic galactooligosaccharides and fructooligosaccharides in combination with *Bifidobacterium breve* M-16V increased serum and intestinal epithelial cell levels of galectin-9 in mice [112]. Infants with atopic dermatitis in a double-blind, placebo-controlled study fed a hydrolysed formula without (control) or with galactooligosaccharides and fructooligosaccharides in combination with *Bifidobacterium breve* for twelve weeks had reduced allergic symptoms [113]. This correlated with increased galectin-9 levels in serum [112]. This indicates that it is possible to increase galectin-9 by dietary administration of pre- and probiotica. However, in a human study, it was not possible to conclude whether galectin-9 levels were protective or promoting for the progression of diabetic nephropathy [114]. Hence, additional studies on galectin-9 and diabetes are needed to see if injection of galectin-9 can prevent diabetes also in human and if the side effects are acceptable.

Galectin-12 was identified in 2001 and is interesting since it is expressed in adipocytes [85, 86]. There are few galectin-12 studies, but importantly galectin-12-negative mice (*Lgals12*^−/−^) [89] have increased lipolysis, adipocyte mitochondrial respiration, reduced whole body lipid content, increased insulin sensitivity compared to wild-type (*Lgals12*^+/+^) mice. Targeting galectin-12 is interesting as it is fat tissue specific and therefore targeting it could help in the prevention/treatment of obesity and diabetes in a targeted manner where other processes, such as cancer, maybe are not affected.

## 3. Conclusions

This review shows that several galectins are key regulators of inflammation in general and that some galectins are involved in the regulation of inflammation processes in obesity leading to, among others, diabetes. Given their important regulatory roles, they are appealing targets for treatment of human diseases. However, limited human studies targeting galectins are available. It is therefore clear that more research is needed to target individual galectins with specificity, in a timely manner and in specific tissues and cells.

## References

[1] Fortuna-Costa A., Gomes A. M., Kozlowski E. O., Stelling M. P., Pavão M. S. G. (2014). Extracellular galectin-3 in tumor progression and metastasis. *Frontiers in Oncology*.

[2] Liu F. T., Patterson R. J., Wang J. L. (2002). Intracellular functions of galectins. *Biochimica et Biophysica Acta (BBA) - General Subjects*.

[3] Chou F. C., Chen H. Y., Kuo C. C., Sytwu H. K. (2018). Role of galectins in tumors and in clinical immunotherapy. *International Journal of Molecular Sciences*.

[4] Pugliese G., Iacobini C., Ricci C., Blasetti Fantauzzi C., Menini S. (2014). Galectin-3 in diabetic patients. *Clinical Chemistry and Laboratory Medicine*.

[5] Stowell S. R., Arthur C. M., Dias-Baruffi M. (2010). Innate immune lectins kill bacteria expressing blood group antigen. *Nature Medicine*.

[6] Stowell S. R., Arthur C. M., McBride R. (2014). Microbial glycan microarrays define key features of host-microbial interactions. *Nature Chemical Biology*.

[7] Wells V., Mallucci L. (1991). Identification of an autocrine negative growth factor: mouse *β*-galactoside-binding protein is a cytostatic factor and cell growth regulator. *Cell*.

[8] Elola M. T., Chiesa M. E., Alberti A. F., Mordoh J., Fink N. E. (2005). Galectin-1 receptors in different cell types. *Journal of Biomedical Science*.

[9] Pereira S. S., Alvarez-Leite J. I. (2014). Low-grade inflammation, obesity, and diabetes. *Current Obesity Reports*.

[10] Ulrich P., Cerami A. (2001). Protein glycation, diabetes, and aging. *Recent Progress in Hormone Research*.

[11] Wentworth J. M., Naselli G., Brown W. A. (2010). Pro-inflammatory CD11c^+^CD206^+^ adipose tissue macrophages are associated with insulin resistance in human obesity. *Diabetes*.

[12] Xu H., Barnes G. T., Yang Q. (2003). Chronic inflammation in fat plays a crucial role in the development of obesity-related insulin resistance. *The Journal of Clinical Investigation*.

[13] Rhodes D. H., Pini M., Castellanos K. J. (2013). Adipose tissue-specific modulation of galectin expression in lean and obese mice: evidence for regulatory function. *Obesity*.

[14] Perillo N. L., Pace K. E., Seilhamer J. J., Baum L. G. (1995). Apoptosis of T cells mediated by galectin-1. *Nature*.

[15] Than N. G., Erez O., Wildman D. E. (2008). Severe preeclampsia is characterized by increased placental expression of galectin-1. *The Journal of Maternal-Fetal & Neonatal Medicine*.

[16] Sturm A., Lensch M., Andre S. (2004). Human galectin-2: novel inducer of T cell apoptosis with distinct profile of caspase activation. *The Journal of Immunology*.

[17] Fuertes M. B., Molinero L. L., Toscano M. A. (2004). Regulated expression of galectin-1 during T-cell activation involves Lck and Fyn kinases and signaling through MEK1/Erk, p38 MAP kinase and p70^s6^ kinase. *Molecular and Cellular Biochemistry*.

[18] Mello C. B., Ramos L., Gimenes A. D., Andrade T. R. . M., Oliani S. M., Gil C. D. (2015). Immunomodulatory effects of galectin-1 on an IgE-mediated allergic conjunctivitis model. *Investigative Ophthalmology & Visual Science*.

[19] Rabinovich G. A., Daly G., Dreja H. (1999). Recombinant galectin-1 and its genetic delivery suppress collagen-induced arthritis via T cell apoptosis. *The Journal of Experimental Medicine*.

[20] Santucci L., Fiorucci S., Cammilleri F., Servillo G., Federici B., Morelli A. (2000). Galectin-1 exerts immunomodulatory and protective effects on concanavalin a–induced hepatitis in mice. *Hepatology*.

[21] Santucci L., Fiorucci S., Rubinstein N. (2003). Galectin-1 suppresses experimental colitis in mice. *Gastroenterology*.

[22] Acar S., Paketçi A., Küme T. (2017). Serum galectin-1 levels are positively correlated with body fat and negatively with fasting glucose in obese children. *Peptides*.

[23] Mukherjee R., Kim S. W., Park T., Choi M. S., Yun J. W. (2015). Targeted inhibition of galectin 1 by thiodigalactoside dramatically reduces body weight gain in diet-induced obese rats. *International Journal of Obesity*.

[24] Kanda A., Dong Y., Noda K., Saito W., Ishida S. (2017). Advanced glycation endproducts link inflammatory cues to upregulation of galectin-1 in diabetic retinopathy. *Scientific Reports*.

[25] Pugliese G., Pricci F., Iacobini C. (2001). Accelerated diabetic glomerulopathy in galectin-3/AGE receptor 3 knockout mice. *The FASEB Journal*.

[26] Christensen M. B., Lawlor D. A., Gaunt T. R. (2006). Genotype of galectin 2 (*LGALS2*) is associated with insulin-glucose profile in the British Women’s Heart and Health Study. *Diabetologia*.

[27] Ozaki K., Inoue K., Sato H. (2004). Functional variation in *LGALS2* confers risk of myocardial infarction and regulates lymphotoxin-*α* secretion in vitro. *Nature*.

[28] Lindskog C., Asplund A., Engkvist M., Uhlen M., Korsgren O., Ponten F. (2010). Antibody-based proteomics for discovery and exploration of proteins expressed in pancreatic islets. *Discovery Medicine*.

[29] Ho M. K., Springer T. A. (1982). Mac-2, a novel 32,000 Mr mouse macrophage subpopulation-specific antigen defined by monoclonal antibodies. *The Journal of Immunology*.

[30] Liu F. T., Albrandt K., Mendel E., Kulczycki A., Orida N. K. (1985). Identification of an IgE-binding protein by molecular cloning. *Proceedings of the National Academy of Sciences of the United States of America*.

[31] Jia S., Wang J. L. (1988). Carbohydrate binding protein 35. Complementary DNA sequence reveals homology with proteins of the heterogeneous nuclear RNP. *Journal of Biological Chemistry*.

[32] Mehul B., Bawumia S., Martin S. R., Hughes R. C. (1994). Structure of baby hamster kidney carbohydrate-binding protein CBP30, an S-type animal lectin. *Journal of Biological Chemistry*.

[33] Fukumori T., Takenaka Y., Yoshii T. (2003). CD29 and CD7 mediate galectin-3-induced type II T-cell apoptosis. *Cancer Research*.

[34] Xue H., Liu L., Zhao Z. (2017). The N-terminal tail coordinates with carbohydrate recognition domain to mediate galectin-3 induced apoptosis in T cells. *Oncotarget*.

[35] Yoshii T., Fukumori T., Honjo Y., Inohara H., Kim H. R. C., Raz A. (2002). Galectin-3 phosphorylation is required for its anti-apoptotic function and cell cycle arrest. *Journal of Biological Chemistry*.

[36] Takenaka Y., Fukumori T., Yoshii T. (2004). Nuclear export of phosphorylated galectin-3 regulates its antiapoptotic activity in response to chemotherapeutic drugs. *Molecular and Cellular Biology*.

[37] Fukumori T., Oka N., Takenaka Y. (2006). Galectin-3 regulates mitochondrial stability and antiapoptotic function in response to anticancer drug in prostate cancer. *Cancer Research*.

[38] Lepur A., Salomonsson E., Nilsson U. J., Leffler H. (2012). Ligand induced galectin-3 protein self-association. *Journal of Biological Chemistry*.

[39] Kuklinski S., Probstmeier R. (1998). Homophilic binding properties of galectin-3: involvement of the carbohydrate recognition domain. *Journal of Neurochemistry*.

[40] von Mach T., Carlsson M. C., Straube T., Nilsson U., Leffler H., Jacob R. (2014). Ligand binding and complex formation of galectin-3 is modulated by pH variations. *Biochemical Journal*.

[41] Straube T., von Mach T., Honig E., Greb C., Schneider D., Jacob R. (2013). Ph-dependent recycling of galectin-3 at the apical membrane of epithelial cells. *Traffic*.

[42] Mazurek N., Conklin J., Byrd J. C., Raz A., Bresalier R. S. (2000). Phosphorylation of the *β*-galactoside-binding protein galectin-3 modulates binding to its ligands. *Journal of Biological Chemistry*.

[43] Balan V., Nangia-Makker P., Kho D. H., Wang Y., Raz A. (2012). Tyrosine-phosphorylated galectin-3 protein is resistant to prostate-specific antigen (PSA) cleavage. *Journal of Biological Chemistry*.

[44] Lepur A., Carlsson M. C., Novak R., Dumic J., Nilsson U. J., Leffler H. (2012). Galectin-3 endocytosis by carbohydrate independent and dependent pathways in different macrophage like cell types. *Biochimica et Biophysica Acta (BBA) - General Subjects*.

[45] Chen H. Y., Fermin A., Vardhana S. (2009). Galectin-3 negatively regulates TCR-mediated CD4^+^ T-cell activation at the immunological synapse. *Proceedings of the National Academy of Sciences of the United States of America*.

[46] Dumic J., Dabelic S., Flögel M. (2006). Galectin-3: an open-ended story. *Biochimica et Biophysica Acta (BBA) - General Subjects*.

[47] Kiwaki K., Novak C. M., Hsu D. K., Liu F. T., Levine J. A. (2007). Galectin-3 stimulates preadipocyte proliferation and is up-regulated in growing adipose tissue. *Obesity*.

[48] Vlassara H., Palace M. R. (2002). Diabetes and advanced glycation endproducts. *Journal of Internal Medicine*.

[49] Iacobini C., Menini S., Ricci C. (2009). Advanced lipoxidation end-products mediate lipid-induced glomerular injury: role of receptor-mediated mechanisms. *The Journal of Pathology*.

[50] Kikuchi Y., Kobayashi S., Hemmi N. (2004). Galectin-3-positive cell infiltration in human diabetic nephropathy. *Nephrology Dialysis Transplantation*.

[51] Fernandes Bertocchi A. P., Campanhole G., Wang P. H. M. (2008). A role for galectin-3 in renal tissue damage triggered by ischemia and reperfusion injury. *Transplant International*.

[52] Martínez-Martínez E., López-Ándres N., Jurado-López R. (2015). Galectin-3 participates in cardiovascular remodeling associated with obesity. *Hypertension*.

[53] Marín-Royo G., Gallardo I., Martínez-Martínez E. (2018). Inhibition of galectin-3 ameliorates the consequences of cardiac lipotoxicity in a rat model of diet-induced obesity. *Disease Models & Mechanisms*.

[54] Paclik D., Danese S., Berndt U., Wiedenmann B., Dignass A., Sturm A. (2008). Galectin-4 controls intestinal inflammation by selective regulation of peripheral and mucosal t cell apoptosis and cell cycle. *PLoS One*.

[55] Hokama A., Mizoguchi E., Sugimoto K. (2004). Induced reactivity of intestinal CD4^+^ T cells with an epithelial cell lectin, galectin-4, contributes to exacerbation of intestinal inflammation. *Immunity*.

[56] Satelli A., Rao P. S., Thirumala S., Rao U. S. (2011). Galectin-4 functions as a tumor suppressor of human colorectal cancer. *International Journal of Cancer*.

[57] Kim S. W., Park K. C., Jeon S. M. (2013). Abrogation of galectin-4 expression promotes tumorigenesis in colorectal cancer. *Cellular Oncology*.

[58] Gendronneau G., Sidhu S. S., Delacour D. (2008). Galectin-7 in the control of epidermal homeostasis after injury. *Molecular Biology of the Cell*.

[59] Gendronneau G., Sanii S., Dang T. (2015). Overexpression of *galectin-7* in mouse epidermis leads to loss of cell junctions and defective skin repair. *PLoS One*.

[60] Sakabe J. I., Kamiya K., Yamaguchi H. (2014). Proteome analysis of stratum corneum from atopic dermatitis patients by hybrid quadrupole-orbitrap mass spectrometer. *The Journal of Allergy and Clinical Immunology*.

[61] Hadari Y. R., Paz K., Dekel R., Mestrovic T., Accili D., Zick Y. (1995). Galectin-8. A new rat lectin, related to galectin-4. *Journal of Biological Chemistry*.

[62] Romaniuk M. A., Tribulatti M. V., Cattaneo V. (2010). Human platelets express and are activated by galectin-8. *Biochemical Journal*.

[63] Bidon N., Brichory F., Bourguet P., Le Pennec J. P., Dazord L. (2001). Galectin-8: a complex sub-family of galectins (review). *International Journal of Molecular Medicine*.

[64] Norambuena A., Metz C., Vicuña L. (2009). Galectin-8 induces apoptosis in Jurkat T cells by phosphatidic acid-mediated ERK1/2 activation supported by protein kinase A down-regulation. *Journal of Biological Chemistry*.

[65] Stowell S. R., Arthur C. M., Slanina K. A., Horton J. R., Smith D. F., Cummings R. D. (2008). Dimeric galectin-8 induces phosphatidylserine exposure in leukocytes through polylactosamine recognition by the C-terminal domain. *Journal of Biological Chemistry*.

[66] Cattaneo V., Tribulatti M. V., Carabelli J., Carestia A., Schattner M., Campetella O. (2014). Galectin-8 elicits pro-inflammatory activities in the endothelium. *Glycobiology*.

[67] Carabelli J., Quattrocchi V., D’Antuono A., Zamorano P., Tribulatti M. V., Campetella O. (2017). Galectin-8 activates dendritic cells and stimulates antigen-specific immune response elicitation. *Journal of Leukocyte Biology*.

[68] Nishi N., Shoji H., Seki M. (2003). Galectin-8 modulates neutrophil function via interaction with integrin *α*M. *Glycobiology*.

[69] Wada J., Kanwar Y. S. (1997). Identification and characterization of galectin-9, a novel *β*-galactoside-binding mammalian lectin. *Journal of Biological Chemistry*.

[70] Wiersma V. R., de Bruyn M., Helfrich W., Bremer E. (2013). Therapeutic potential of galectin-9 in human disease. *Medicinal Research Reviews*.

[71] Kashio Y., Nakamura K., Abedin M. J. (2003). Galectin-9 induces apoptosis through the calcium-calpain-caspase-1 pathway. *The Journal of Immunology*.

[72] Chen X., Song C. H., Liu Z. Q. (2011). Intestinal epithelial cells express galectin-9 in patients with food allergy that plays a critical role in sustaining allergic status in mouse intestine. *Allergy*.

[73] Katoh S., Ishii N., Nobumoto A. (2007). Galectin-9 inhibits CD44–hyaluronan interaction and suppresses a murine model of allergic asthma. *American Journal of Respiratory and Critical Care Medicine*.

[74] Miko E., Meggyes M., Bogar B. (2013). Involvement of Galectin-9/TIM-3 pathway in the systemic inflammatory response in early-onset preeclampsia. *PLoS One*.

[75] Sun J., Yang M., Ban Y. (2016). Tim-3 is upregulated in NK cells during early pregnancy and inhibits NK cytotoxicity toward trophoblast in galectin-9 dependent pathway. *PLoS One*.

[76] Koopman L. A., Kopcow H. D., Rybalov B. (2003). Human decidual natural killer cells are a unique NK cell subset with immunomodulatory potential. *The Journal of Experimental Medicine*.

[77] Lachapelle M. H., Miron P., Hemmings R., Roy D. C. (1996). Endometrial T, B, and NK cells in patients with recurrent spontaneous abortion. Altered profile and pregnancy outcome. *The Journal of Immunology*.

[78] Tang Z. H., Liang S., Potter J., Jiang X., Mao H. Q., Li Z. (2013). Tim-3/galectin-9 regulate the homeostasis of hepatic NKT cells in a murine model of nonalcoholic fatty liver disease. *The Journal of Immunology*.

[79] Chou F. C., Shieh S. J., Sytwu H. K. (2009). Attenuation of Th1 response through galectin-9 and T-cell Ig mucin 3 interaction inhibits autoimmune diabetes in NOD mice. *European Journal of Immunology*.

[80] Rodrigues-Alcazar J. F., Ataide M. A., Engels G. (2018). Charcot-Leyden crystals activate the NLRP3 inflammasome and cause IL-1*β* inflammation. *bioRxiv*.

[81] Dvorak A. M., Letourneau L., Login G. R., Weller P. F., Ackerman S. J. (1988). Ultrastructural localization of the Charcot-Leyden crystal protein (lysophospholipase) to a distinct crystalloid-free granule population in mature human eosinophils. *Blood*.

[82] Kubach J., Lutter P., Bopp T. (2007). Human CD4^+^CD25^+^ regulatory T cells: proteome analysis identifies galectin-10 as a novel marker essential for their anergy and suppressive function. *Blood*.

[83] Lingblom C., Andersson J., Andersson K., Wenneras C. (2017). Regulatory eosinophils suppress T cells partly through galectin-10. *The Journal of Immunology*.

[84] Choi E., Miller A. D., Devenish E., Asakawa M., McConkey M., Peters-Kennedy J. (2017). Charcot–Leyden crystals: do they exist in veterinary species? A case report and literature review. *Journal of Veterinary Diagnostic Investigation*.

[85] Yang R. Y., Hsu D. K., Yu L., Ni J., Liu F. T. (2001). Cell cycle regulation by galectin-12, a new member of the galectin superfamily. *Journal of Biological Chemistry*.

[86] Hotta K., Funahashi T., Matsukawa Y. (2001). Galectin-12, an adipose-expressed galectin-like molecule possessing apoptosis-inducing activity. *Journal of Biological Chemistry*.

[87] Wan L., Yang R.-Y., Liu F.-T. (2018). Galectin-12 in cellular differentiation, apoptosis and polarization. *International Journal of Molecular Sciences*.

[88] Lo C.-W., Chen C. S., Chen Y. C. (2018). Allyl isothiocyanate ameliorates obesity by inhibiting galectin-12. *Molecular Nutrition & Food Research*.

[89] Yang R. Y., Yu L., Graham J. L. (2011). Ablation of a galectin preferentially expressed in adipocytes increases lipolysis, reduces adiposity, and improves insulin sensitivity in mice. *Proceedings of the National Academy of Sciences of the United States of America*.

[90] Wan L., Lin H. J., Huang C. C. (2016). Galectin-12 enhances inflammation by promoting M1 polarization of macrophages and reduces insulin sensitivity in adipocytes. *Glycobiology*.

[91] Odegaard J. I., Ricardo-Gonzalez R. R., Red Eagle A. (2008). Alternative M2 activation of Kupffer cells by PPAR*δ* ameliorates obesity-induced insulin resistance. *Cell Metabolism*.

[92] Pang J., Rhodes D. H., Pini M. (2013). Increased adiposity, dysregulated glucose metabolism and systemic inflammation in galectin-3 KO mice. *PLoS One*.

[93] Than N. G., Romero R., Goodman M. (2009). A primate subfamily of galectins expressed at the maternal–fetal interface that promote immune cell death. *Proceedings of the National Academy of Sciences of the United States of America*.

[94] Than N. G., Pick E., Bellyei S. (2004). Functional analyses of placental protein 13/galectin-13. *European Journal of Biochemistry*.

[95] Than N. G., Abdul Rahman O., Magenheim R. (2008). Placental protein 13 (galectin-13) has decreased placental expression but increased shedding and maternal serum concentrations in patients presenting with preterm pre-eclampsia and HELLP syndrome. *Virchows Archiv*.

[96] Sekizawa A., Purwosunu Y., Yoshimura S. (2009). PP13 mRNA expression in trophoblasts from preeclamptic placentas. *Reproductive Sciences*.

[97] Fritz R., Kohan-Ghadr H. R., Bolnick J. M. (2015). Noninvasive detection of trophoblast protein signatures linked to early pregnancy loss using trophoblast retrieval and isolation from the cervix (TRIC). *Fertility and Sterility*.

[98] Bolnick J. M., Kohan-Ghadr H. R., Fritz R. (2016). Altered biomarkers in trophoblast cells obtained noninvasively prior to clinical manifestation of perinatal disease. *Scientific Reports*.

[99] Blanchard H., Bum-Erdene K., Bohari M. H., Yu X. (2016). Galectin-1 inhibitors and their potential therapeutic applications: a patent review. *Expert Opinion on Therapeutic Patents*.

[100] Collins P. M., Öberg C. T., Leffler H., Nilsson U. J., Blanchard H. (2012). Taloside inhibitors of galectin-1 and galectin-3. *Chemical Biology & Drug Design*.

[101] Perone M. J., Bertera S., Shufesky W. J. (2009). Suppression of autoimmune diabetes by soluble galectin-1. *The Journal of Immunology*.

[102] Lin Y.-T., Chen J. S., Wu M. H. (2015). Galectin-1 accelerates wound healing by regulating the neuropilin-1/Smad3/NOX4 pathway and ROS production in myofibroblasts. *Journal of Investigative Dermatology*.

[103] Wu X., Li J., Connolly E. M. (2017). Combined anti-VEGF and anti–CTLA-4 therapy elicits humoral immunity to galectin-1 which is associated with favorable clinical outcomes. *Cancer Immunology Research*.

[104] Nguyen M.-N., Su Y., Kiriazis H. (2018). Upregulated galectin-3 is not a critical disease mediator of cardiomyopathy induced by *β*_2_-adrenoceptor overexpression. *American Journal of Physiology-Heart and Circulatory Physiology*.

[105] Vergaro G., Prud’homme M., Fazal L. (2016). Inhibition of galectin-3 pathway prevents isoproterenol-induced left ventricular dysfunction and fibrosis in mice. *Hypertension*.

[106] Sathisha U. V., Jayaram S., Harish Nayaka M. A., Dharmesh S. M. (2007). Inhibition of galectin-3 mediated cellular interactions by pectic polysaccharides from dietary sources. *Glycoconjugate Journal*.

[107] Stegmayr J., Lepur A., Kahl-Knutson B. (2016). Low or no inhibitory potency of the canonical galectin carbohydrate-binding site by pectins and galactomannans. *Journal of Biological Chemistry*.

[108] Blanchard H., Yu X., Collins P. M., Bum-Erdene K. (2014). Galectin-3 inhibitors: a patent review (2008–present). *Expert Opinion on Therapeutic Patents*.

[109] Harrison S. A., Marri S. R., Chalasani N. (2016). Randomised clinical study: GR-MD-02, a galectin-3 inhibitor, vs. placebo in patients having non-alcoholic steatohepatitis with advanced fibrosis. *Alimentary Pharmacology & Therapeutics*.

[110] Ritchie S., Neal D., Shlevin H., Allgood A., Traber P. (2017). A phase 2a, open-label pilot study of the galectin-3 inhibitor GR-MD-02 for the treatment of moderate-to-severe plaque psoriasis. *Journal of the American Academy of Dermatology*.

[111] Kanzaki M., Wada J., Sugiyama K. (2012). Galectin-9 and t cell immunoglobulin mucin-3 pathway is a therapeutic target for type 1 diabetes. *Endocrinology*.

[112] de Kivit S., Saeland E., Kraneveld A. D. (2012). Galectin-9 induced by dietary synbiotics is involved in suppression of allergic symptoms in mice and humans. *Allergy*.

[113] van der Aa L. B., Heymans H. S., van Aalderen W. M. (2010). Effect of a new synbiotic mixture on atopic dermatitis in infants: a randomized-controlled trial. *Clinical & Experimental Allergy*.

[114] Kurose Y., Wada J., Kanzaki M. (2013). Serum galectin-9 levels are elevated in the patients with type 2 diabetes and chronic kidney disease. *BMC Nephrology*.

